# Synthesis, Structural Analysis, and Biological Activities of Some Imidazolium Salts

**DOI:** 10.1155/2018/1439810

**Published:** 2018-05-22

**Authors:** Gühergül Uluçam, Murat Turkyilmaz

**Affiliations:** Department of Chemistry, Trakya University, 22030 Edirne, Turkey

## Abstract

Four newly synthesized imidazolium salts were characterized by nuclear magnetic resonance, vibrational spectra, and mass spectra. Then, the density functional theory calculations were performed to obtain the molecular configurations on which the theoretical nuclear magnetic resonance and infrared spectra were consequently obtained. The comparison of calculated spectra with the experimental spectra for each molecule leads to the conclusion that the theoretical results can be assumed to be a good approach to their molecular configurations. The in vitro biological activities of the salts on the selected bacteria and cancer cell lines were determined by using the broth dilution method according to Clinical and Laboratory Standards Institute guidelines. The 1,3-bis(2-hydroxyethyl) imidazolidinium bromide and 3-(2-ethoxy-2-oxoethly)-1-(3-aminopropyl)-1H-imidazol-3-ium bromide showed efficiency on *Bacillus cereus* ATCC 11778. The 3-bis(2-carboxyethyl)-4-methyl-1-H-imidazol-3-ium bromide was effective on HeLa while a similar effect was observed on Hep G2 with 3-(2-carboxyethyl)-1-(3-aminopropyl)-1H-imidazol-3-ium bromide.

## 1. Introduction

Imidazole rings are building blocks in amino acids [1] together with the fact that their membership in the development of new antifungal drugs [2, 3] and antibiotics [4, 5] are crucial. Its derivatives are widely used in other medicinal applications [6]. The pregnane derivatives with imidazole moiety and triazole moiety, for example, were tested on the prostate, breast, and lung cancer cell lines, and dose-effective proliferation of the cells was determined [7]. Similarly, the novel hybrid compounds of imidazole scaffold-based 2-benzylbenzofloran have been prepared and used in cytotoxic activity studies on various cancer cell lines [8].

As a five-membered aromatic ring containing two nonadjacent nitrogen atoms [9], imidazole is also subjected to various computational chemistry research beyond its biological applications. Its ability to capture CO_2_ was determined in the investigations of the greenhouse effect compensation in the framework of van der Waals bonded host-guest relation [10]. The hydroxyl conductivity in polymembranes based on imidazole salts was simulated using radial distribution functions and found that the imidazole groups provide better conductivity than that of water and methanol [11]. Moreover, the specific imidazole derivatives exhibited good cross section values for two-photon absorption [12]. The detoxification of phosphotriesters by imidazole rings was clarified comparing the same effects with methylimidazoles depending on the methyl positioning [13].

*N*-Heterocyclic carbenes (NHCs) are the imidazole-based carbene groups which are isolated and crystallized by the deprotonation of imidazole salts [14]. Also, the imidazole salts naturally transform into NHCs over metal complex building reactions, as exampled on the synthesis and characterization of the silver-NHC complexes [15] and the iron-imidazole salts [16].

The constitution and functions of imidazole ligands in organometallic chemistry and inorganic chemistry have been widely studied, and these particular researches have been evaluated as a scientific competition field due to its importance in the related industry [17]. Also, they are set as alternative to usual ligands in the carbon-carbon coupling reactions of the pharmaceutical reagents [18]. The synthesis and spectroscopic characterizations of four new NHC ligands, namely, *1,3-bis(2-hydroxyethyl) imidazolidinium bromide* (*L*_A_), *3-(2-ethoxy-2-oxoethly)-1-(3-aminopropyl)-1H-imidazol-3-ium bromide* (*L*_B_), *1,3-bis(2-carboxyethyl)-4-methyl-1H-imidazol-3-ium bromide* (*L*_C_), and *3-(2-carboxyethyl)-1-(3-aminopropyl)-1H-imidazol-3-ium bromide* (*L*_D_) were exhibited in this study. Using their nuclear magnetic resonance (NMR) and infrared (IR) spectra, the molecular properties of the ligands were obtained. Also, the *in vitro* biological activities of the synthesized molecules were presented.

## 2. Materials and Methods

### 2.1. Instrumentation and Methods

The ^1^H and ^13^C NMR spectra of the compounds in deuterium oxide (D_2_O) were recorded on Varian 300 MHz and Varian 75.5 MHz, respectively. The IR spectra by KBr pellets were recorded in the range 450–4000 cm^−1^ by a PerkinElmer BXII spectrometer. The mass spectra were acquired by the electron impact technique using a Thermo Finnegan Trace DSQ GC/MS. Elemental analyses for C, H, and N were realized on the dried samples using a PerkinElmer 2400 CHN analyzer. The absorbance measurements in determining the biological activities of the material were carried out in Thermo Scientific Multiskan Go multiplate spectrophotometer.

### 2.2. Synthesis

#### 2.2.1. 1,3-Bis(2-hydroxyethyl) Imidazolidinium Bromide, *L*_A_

Imidazole (10 mmol, 0.68 g) was dissolved in tetrahydrofuran (THF), and bromoethanol (22 mmol, 2.75 g) was added as the mixture was stirred for 20 hours. The completion of the reaction was monitored by thin-layer chromatography (TLC) in ethyl acetate/hexane (1 : 5) analyses, and the solid residue was filtered out with a sintered glass funnel. The solvent in the filtrate was evaporated using a rotary evaporator, and the product was dried in a vacuum desiccator. After that, the product was purified by column chromatography (ethyl acetate/hexane, 1 : 5). The best yield was obtained when the reaction was carried out at room temperature with a 1 : 2 mole ratio of the reagents. 1.54 g of the final product was obtained with 65% yield. It was in yellowish liquid form. The elemental analyses result for *L*_A_ with the chemical formula C_7_H_13_BrN_2_O_2_ are C, 35.46%; H, 5.53%; and N, 11.82%; found: C, 35.35%; H, 5.43%; and N, 11.73%. The mass spectroscopy reads (*m/z*) 158.22 (M + H)^+^ which is consistent with the expected molecular weight.

#### 2.2.2. 3-(2-Ethoxy-2-oxoethly)-1-(3-aminopropyl)-1H-imidazol-3-ium Bromide, *L*_B_

3-(1*H*-imidazol-1-yl)propan-1-amine (10 mmol, 1.27 g) was dissolved in THF and stirred at room temperature, and then, ethyl bromoacetate (11 mmol, 1.837 g) was added with a 1 : 1 mole ratio. The mixture was stirred for 15 hr. The completion of the reaction was monitored by thin layer chromatography (TLC) in ethyl acetate/hexane (1 : 5) analyses. The solvent was evaporated in a rotary evaporator, and the substance was kept under vacuum in a desiccator. After that, the product was purified by column chromatography (ethyl acetate/hexane, 1 : 5). It was pale brown oily liquid. 0.94 g of the final product was obtained with 32% yield. The elemental analyses result for *L*_B_ with the chemical formula C_10_H_18_BrN_3_O_2_ are C, 41.11%; H, 6.21%; and N, 14.38%; found: C, 39.93%; H, 6.35%; N, and 14.21%. The mass spectroscopy result reads (*m/z*) 213.12 (M  + H)^+^ which is consistent with the expected molecular weight.

#### 2.2.3. 1,3-Bis(2-carboxyethyl)-4-methyl-1H-imidazol-3-ium Bromide, *L*_C_

The procedure applied in the synthesis of *L*_A_ was also used for the synthesis of *L*_C_ by replacing imidazole and bromoethanol with 4-methylimidazole (10 mmol, 0.82 g) and 3-bromopropionic acid (22 mmol, 3.366 g), respectively. After that, the product was purified by column chromatography (ethyl acetate/hexane, 1 : 5). It was in white solid form. 1.48 g of the final product was obtained with 48% yield. The elemental analyses result for *L*_C_ with the chemical formula C_10_H_15_BrN_2_O_4_ are C, 39.10%; H, 4.92%; and N, 9.12%; found: C, 38.97%; H, 4.77%; and N, 19.22%. The mass spectroscopy result reads (*m/z*) 227.68 (M + H)^+^ which is consistent with the expected molecular weight.

#### 2.2.4. 3-(2-Carboxyethyl)-1-(3-aminopropyl)-1H-imidazol-3-ium Bromide, *L*_D_

The procedure applied in the synthesis of *L*_B_ was also used for the synthesis of *L*_D_ by replacing ethyl bromoacetate with 3-bromopropanoic acid (11 mmol, 1.683 g), respectively. The product was purified by column chromatography (ethyl acetate/hexane, 1 : 5). It was pale brown oily liquid. 1.22 g of the final product was obtained with 44% yield. The elemental analyses result for *L*_D_ with the chemical formula C_9_H_16_BrN_3_O_3_ are C, 38.86%; H.5.80%; and N, 15.11%; found: C, 38.77%; H, 5.63%; and N, 15.23%. The mass spectroscopy result reads (*m/z*) 198.25 (M + H)^+^ which is consistent with the expected molecular weight.

### 2.3. Biological Activities

#### 2.3.1. Antibacterial Activity

Broth microdilution method as in the guidelines of Clinical Laboratory Standards Institute was applied to determine the antibacterial activities of the *L*_A_, *L*_B_, *L*_C_, and *L*_D_ on the Gram-negative bacteria (*Escherichia coli* ATCC 25922, *Escherichia coli* 0157:H7, and *Salmonella typhimurium* ATCC 14028), Gram-positive bacteria (*Bacillus cereus* ATCC 11778, *Staphylococcus aureus* ATCC 25923, and *Listeria monocytogenes* ATCC 19115), and standard yeast *Candida albicans* ATCC 10231. The bacteria and yeast were obtained from the American Type Culture Collection. The incubations were done in Tryptic Soy Broth medium at 37°C for 24 hr in the scale of McFarland 0.5. The antibiotic controls were carried by gentamicin on *Bacillus cereus* ATCC 1177 and amphotericin-b on *Candida albicans* ATCC 10231, while ampicillin was used on the other bacteria samples. For sterilization purposes, the antibiotic solutions and the stock solution of the chemicals were filtered through a 0.45 *μ*m sterile filter. The solvent in which the test compounds dissolved was dimethyl sulfoxide (DMSO) which did not show any inhibition effect on the bacteria. The pure *L*_A_, *L*_B_, *L*_C_, and *L*_D_ solutions and the pure microorganism planted mediums were used as the sterility and the growth controls, respectively. The six different concentrations of the each compound were applied to the cells starting from 32 *μ*m and diluting to half each time. Each of the 96 microplates was planted in 150 *μ*l of Tryptic Soy Broth medium, 30 *μ*l of the bacteria or the yeast culture, and 20 *μ*l of the chemical compound solution. All microplates were incubated at 37°C for 24 hr. The absorbance was measured at 600 nm.

#### 2.3.2. Cytotoxicity

3-(4,5-Dimethylthiazol-2-yl)-2,5-diphenyltetrazolium bromide (MTT) assay was used to observe the cytotoxicity of synthesized compounds on human cervical cancer cell line (HeLa), human liver cancer cell line (Hep G2), and healthy mouse embryonic fibroblast cell line (MEF). All cell lines were provided with American Type Culture Collection (ATCC, Manassas, VA, USA). A certain population of the cells were incubated in Dulbecco's modified Eagle's Medium (DMEM) provided with Life Technologies GIBCO, Grand Island, NY, USA involving 1% penicillin streptomycin and 1% L-glutamine and HAMS F12 (1 : 1) broth medium at 37°C under 5% CO_2_. The cells were planted in 96-multiwell plates with approximately equal numbers of 10^5^, and they were allowed further incubation for 24 hr. The seven different doses of each compound were applied to the cancer cell lines and the MEF cells. The applied doses were 400 *μ*m, 200 *μ*m, 100 *μ*m, 50 *μ*m, 25 *μ*m, 12.5 *μ*m, and 6.25 *μ*m, and the dose application time was 24 hr. The surviving control for each cell was carried out against the cells not exposed to any dose. Then, 20 *μ*l/plate of 5 mg/ml MTT solution was added into the each well and left to further incubation between 2 and 4 hr. The excess MTT solutions were removed from the wells, and 200 *μ*l of ultra-pure DMSO was added. The set was left in dark for 5 min before measuring the color intensities on a 492 nm spectrophotometer.

### 2.4. Computational Modeling

All calculations were carried out in the framework of Gaussian 09 package. The molecules were optimized in their ground state using Becke3-Lee-Yang-Parr (B3LYP) exchange correlation functional method and 6-311G + (2d, p) basis set within density functional theory (DFT). Then, the NMR spectra and the IR spectra were calculated on the optimized geometries using the same method and the same basis set. The gauge-independent atomic orbital (GIAO) method was adopted to acquire theoretical ^1^H and ^13^C NMR shifts, which were converted to that of tetramethylsilane scale. The IR spectra were scaled by the factor 0.9613 due to the theoretical miscalculations [19–21]. The vibrational modes were assigned by observing the animation property of the frequency calculations provided with Gaussian 09 package.

## 3. Result and Discussion

NMR and IR spectra are reliable methods to elucidate an organic material or some of metal complexes. They can also be used to verify calculated molecular structure by comparing calculated spectra with corresponding experimental spectra and thus determining structural parameters and complete description of chemicals investigated.

### 3.1. Molecular Structures

The NMR measurements were taken in dilute D_2_O solution, while the IR spectra were recorded in the solid KBr pellet. Therefore, NMR and infrared spectra may reflect different molecular structures as the molecule surrounded by D_2_O molecules in NMR spectra while the intermolecular interactions exist in the IR spectra, especially on -CH and -OH bonds [22]. On the theoretical calculation side, the crystalline phase calculations were excluded due to the single crystal form of the molecules obtained for X-ray analysis. The gas phase calculations were adopted considering differences with other phases with 2% maximum error margin which were especially on the -CH and -OH bond lengths.

**Scheme 1:**
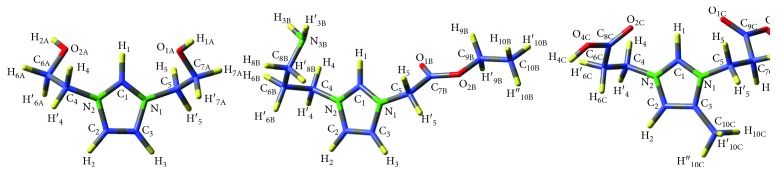
Optimized conformers of the synthesized imidazole molecules with their labeled and numbered atoms: (a) 1,3-bis(2-hydroxyethyl) imidazolidinium bromide, (b) 3-(2-ethoxy-2-oxoethly)-1-(3-aminopropyl)-1H-imidazol-3-ium bromide, (c) 1,3-bis(2-carboxyethyl)-4-methyl-1H-imidazol-3-ium bromide, and (d) 3-(2-carboxyethyl)-1-(3-aminopropyl)-1H-imidazol-3-ium bromide.

The process of the theoretical modeling of the molecules has initially been realized by using the potential energy surface scanning method for the selected dihedral angles of each molecule, thus obtaining the lowest energy conformers in the gas phase, and the resulting optimized conformers are given in Scheme 1. The imidazole rings except that of *L*_C_ with its CH_3_ attachment and the CH_2_ groups directly bonded to the nitrogen of the rings are common in the all molecules.

*L*_A_ has the point group symmetry C2 as the other molecules were found to have C1 symmetry. The lower point group symmetry of the *L*_B_, *L*_C_, and *L*_D_ in comparison with the C2 symmetry of *L*_A_ is because of the attachment of the different aliphatic chains to their imidazole rings. The calculated optimized energies and the dipole moments of *L*_A_, *L*_D_, *L*_B_, and *L*_C_ are, respectively, −534.4, −667.3, −706.6, and −800.5 in units of a.u. and 2.11, 3.41, 4.54, and 7.78 in units of Debye.

The calculated parameters of the common properties of the molecules are presented in Table 1. Although the molecules differ from each other by their moieties bonded to the imidazole rings, the bond lengths, the bond angles, and the dihedral angles belong to the ring, and its immediate vicinity are in good agreement with each other and with the corresponding X-ray diffractometer (XRD) results of previously investigated similar molecule *1,3-bis(acetamide)imidazol-3-ium bromide* which was crystallized successfully [22]. The dihedral angles chosen on the imidazole ring are either about 0° or 180°, implying the aromatic structure as expected. The C-N bond lengths which bind the rings and the aliphatic moieties, that is, C_4_-N_2_ and N_1_-C_5_ are equal in *L*_A_ reflecting the symmetric structure of the molecule. However, these are different in *L*_B_, *L*_C_, and *L*_D_ because of the different moieties on the both side of their aromatic rings. Although the symmetry of *L*_C_ was broken by the C-CH_3_ group instead of C-H in the ring, no drastic changes were observed in the concerning ring parameters. It can be inferred that the common geometrical parameters are consistent with each other with a priori theoretical confirmations before NMR and IR spectra clarifications of the calculated molecular structures.

### 3.2. Nuclear Magnetic Resonance Spectra

The experimental and theoretical chemical shifts of ^1^H NMR and ^13^C NMR spectra of *L*_A_, *L*_B_, *L*_C_, and *L*_D_ molecules are given in Table 2. The calculations were carried out in the gas phase considering their optimized geometries given in Scheme 1. The proton signals for -NH_2_ and -OH in all were absent in the experimental ^1^H NMR spectra because the solvent was D_2_O concerning the solubility of the specimens which exchanged NH_2_ and OH protons with deuterium. Although their detailed analysis left to the IR spectra in the following section, the calculated shifts *δ* 1.73 ppm of OH in *L*_A_ and *δ* 0.5 ppm and *δ* 0.7 ppm of NH_2_ in *L*_B_ and *L*_D_, respectively, are in the typical chemical shift range of R-NH_2_ and R-OH groups.

The imidazole proton (NCHN, H_1_) shifts of *L*_A_, *L*_C_, and *L*_D_ with 9.06 ppm, 8.84 ppm, and 9.11 ppm, respectively, are noticeably bigger than that of *L*_B_ with 7.79 ppm. That can be due to the intramolecular interactions of the NCHN hydrogen with the close oxygen atoms since the oxygen reduces the electron density on the hydrogen depending on the distance between them, thus causing higher NMR shifts. Indeed, the H_1_-O_1A_ in *L*_A_, the H_1_-O_1C_ in *L*_C_, and the H_1_-O_1D_ in *L*_D_ are 2.61 Å, 2.42 Å, and 2.41 Å, respectively. These distances which were measured from the theoretical models of the molecules are quite longer than that 2.85 Å distance of the H_1_-O_1B_ measured in *L*_B_ which results in small shifts for the H_1_ of *L*_B_. This is supported by the fact that the other imidazole proton shifts of H_2_ and H_3_ are all in close values about 7.7 ppm for all molecules as no distinct intermolecular interactions possible on H_2_ and H_3_ in any of them. In addition, the NMR signals of CH_2_ protons in the aliphatic chains of all molecules and the smallest proton shifts of CH_3_ protons in *L*_B_ and *L*_C_ molecules are theoretically and experimentally in agreement with the expected NMR results. These NMR data are in accordance with the previous study on a similar imidazole salt [23, 24] in which the protons belong to the aliphatic chain found in 4.15 ppm–2.08 ppm, the carbons belong to the imidazole ring, and the aliphatic chain is found in 139.2 ppm–123 ppm and 62.5 ppm–33.9 ppm as in this study.

The eight *R*-squared tests using the data in Table 2 provide least 99.8% agreement between the experimental and the theoretical ^1^H and ^13^C NMR for *L*_A_, *L*_B_, *L*_C_, and *L*_D_. The theoretical results for NMR are in very good agreement with the experimental results as well as the observation of the expected specific values. Thus, one can infer that the calculated atomic configurations of the all molecules are good estimations except the exclusion of the interchangeable hydrogens.

### 3.3. Infrared Spectra

**Figure 1:**
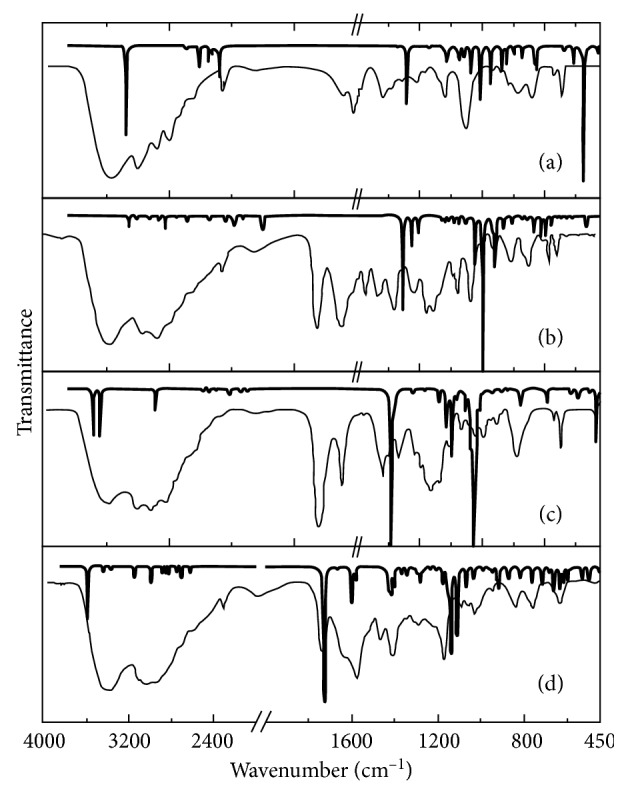
Calculated (bold lines) and experimental (pale lines) infrared spectra of the synthesized imidazole molecules: (a) *1,3-bis(2-hydroxyethyl) imidazolidinium bromide*, (b) *3-(2-ethoxy-2-oxoethly)-1-(3-aminopropyl)-1H-imidazol-3-ium bromide*, (c) *1,3-bis(2-carboxyethyl)-4-methyl-1H-imidazol-3-ium bromide*, and (d) *3-(2-carboxyethyl)-1-(3-aminopropyl)-1H-imidazol-3-ium bromide*.

In Figure 1, the experimental infrared spectra of *L*_A_, *L*_B_, *L*_C_, and *L*_D_ in the 450–4000 cm^−1^ region are given against their IR spectra calculations. The detailed account of the IR spectra including the in-plane vibrations of the stretching, scissoring, and rocking and the out-of-plane vibrations of the wagging and twisting for each molecule is presented for *L*_A_, *L*_B_, *L*_C_, and *L*_D_ in Tables 3 [Table tab4] [Table tab5]–6, respectively. The *R*-square test results exhibit 99.8%, 99.5%, 99.5%, and 99.7% agreement between the experimental and the theoretical IR spectra of *L*_A_, *L*_B_, *L*_C_, and *L*_D_, respectively.

The intermolecular interactions and the correlations of close frequency IR signals cause the -OH, -CH, -CH_2_, -CH_3_, and -NH_2_ stretching vibrations to appear under broad peaks in the experimental spectra. These broad peaks are in very well-defined frequency region. The vibrations coming from the other functional groups of the imidazole rings and the aliphatic chains including the other vibrations of the hydrogenic groups in all modes appeared in 1750–450 cm^−1^ region.

The first vibration signals observed in the 1800–1700 cm^−1^ region of the experimental spectra of *L*_B_, *L*_C_, and *L*_D_ are distinct peaks arising from ν(C=O) stretching as their values in agreement with the previously observed ν(C=O) stretching [25] and theoretically calculated values as reflected in Table 3. The imidazole ν(C=N) stretching of *L*_A_ appears as individual signals while they are coupled under broad peaks with *L*_B_, *L*_C_, and *L*_D_ molecules as seen about 1650 cm^−1^ while the frequencies for ν(C=N) stretching are in agreements with the corresponding data as given in [26]. The aliphatic ν(C-N) stretching in 1069–1235 cm^−1^ interval and the aliphatic ν(C-C) stretching in 917–1099 cm^−1^ are consistent with the previous corresponding measurements [27].

Beyond the consistency of the common imidazole properties of the molecules, we also give unique infrared signals of molecules in Table 3. C-O stretching belong to the H_2_C-OH group in *L*_A_, in-plane *δ* vibrations of CH_3_ at the end of the aliphatic chain of *L*_B_, and at the attachment of the imidazole ring of *L*_C_ are such unique vibrations. The calculated and observed wave numbers of these vibrations are consistent with the concerning previous studies [28, 29].

The molecules in consideration analyzed by infrared spectrum because of the lack of exchangeable protons of -OH and -NH_2_ in the NMR spectra of the chemicals and the existence of these groups is proved in the molecules. Also, the IR spectra of the molecules reverifies the theoretically obtained configurations of them as they were first verified by the comparison the theoretical NMR spectra with that of the experimental NMR spectra.

### 3.4. Antibacterial and Cytotoxic Activities

**Figure 2:**
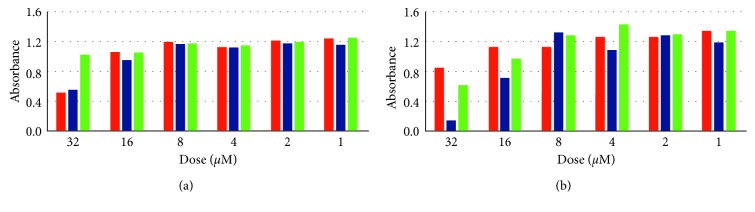
(a) The antibacterial activity on *Candida albicans*. The first bars on the left are for the commercial antibiotic inhibition for each dose. The second bars are for *1,3-bis(2-hydroxyethyl) imidazolidinium bromide* (*L*_A_), and the third bars are for *3-(2-ethoxy-2-oxoethly)-1- (3-aminopropyl)-1H-imidazol-3-ium bromide* (*L*_B_), (b) same as in Figure 2(a), but for the antibacterial activity on *Bacillus cereus*.

**Figure 3:**
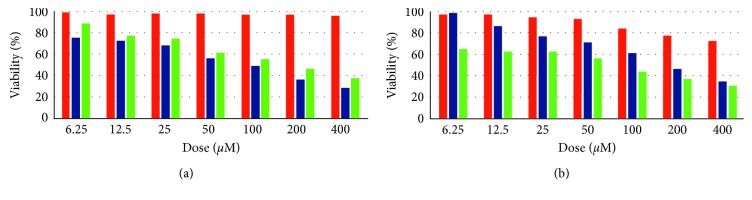
(a) The cytotoxicity of *1,3-bis(2-carboxyethyl)-4-methyl-1H-imidazol-3-ium bromide*. The first bars represent the effect on healthy mouse embryonic fibroblast cell line for each dose, as do the second bars on human cervical cancer cell line, and the third bars on human liver cancer cell line, (b) same as in Figure 3(a), but for the cytotoxicity of *3-(2-carboxyethyl)-1-(3-aminopropyl)-1H-imidazol-3-ium bromide*.

The antibacterial tests of the synthesized four molecules were run on the six different bacteria and yeast, as mentioned in Section 2.3.1. Figures 2(a) and 3(b) show the inhibitory effects of the various concentrations of the *L*_A_ and *L*_B_ on *Candida albicans* ATCC 10231 and *Bacillus cereus* ATCC 11778 (Gram positive) together with that of the antibiotic controls. Their effects on *Escherichia coli* O157:H7 (Gram negative), *Escherichia coli* ATCC 25922, *Salmonella typhimurium* ATCC 14028, *Staphylococcus aureus* ATCC 25923, and *Listeria monocytogenes* ATCC 19115 are excluded because of their very weak efficacy in comparison with that of the antibiotic control. Although the absorbance measurements involve some statistical errors, *L*_A_ showed better inhibition than the antibiotic on *Bacillus cereus* ATCC 11778, while both chemicals are effective on the selected bacteria and yeast as much as the antibiotics. *L*_C_ and *L*_D_ show no noticeable inhibitory effects on the target bacteria, and thus, their absorbance values as function of their concentration were not given for the sake of brevity.

Figure 3(a) shows the cytotoxic activity of *L*_C_ on HeLa and Hep G2 cell lines against healthy MEF cell lines, as does Figure 3(b) for the cytotoxic activity of *L*_D_. The cytotoxic activities of *L*_A_ and *L*_B_ are not exhibited as they showed lesser cytotoxic activities in comparison with *L*_C_ and *L*_D_. The percentage cell viability of HeLa and Hep G2 cell lines was significantly reduced by *L*_C_ and *L*_D_ at the end of 24 hr application of the doses. In addition, *L*_C_ did not harm the healthy MEF cell lines for any dose so that its half inhibitory concentrations (IC_50_) cannot be calculated. Meantime, *L*_D_ showed some activity on the MEF cells with the high doses together with the fact that the activity was not as strong as it did on the cancer cell lines.

The antibacterial and cytotoxic effectiveness of the synthesized molecules are summarized in Table 7 by presenting their IC_50_ concentrations in the units of *µ*M on the bacteria together with the IC_50_ values of the antibiotics and on the cancer cell lines. The lack of the IC_50_ values of *L*_C_ and *L*_D_ for antibacterial activity in Table 7 indicates that the IC_50_ values of them cannot be calculated due to their very weak effect on the bacteria sample within the dose range considered. *L*_A_ and *L*_B_ were, respectively, thrice and twice more effective on *Bacillus cereus* than the antibiotic (gentamicin) as the *L*_A_ equals the antibiotic (amphotericin-b) effect on *Candida albicans*.

The cell viability assay of the chemicals exhibits no harmful effect on the healthy MEF cell lines as their IC_50_ values cannot be calculated within the dose range considered. The *L*_C_ inhibition on HeLa and the *L*_D_ inhibition on Hep G2 are distinctive when they are compared with the inhibition of the other imidazole-based chemicals on different cancer cell lines in the concerning studies. The IC_50_ values of the *L*_C_ on HeLa and the *L*_D_ on Hep G2 were 81 *µ*M and 57 *µ*M, respectively. The similar imidazole compounds which have alkyl moieties were tested on the cancer cells different from the cells used in this study [8]. The IC_50_ values of *1-(benzofuran-2-yl(phenyl)methyl)-3-allyl-2-ethyl-1H-imidazol-3-ium bromide* and *1-(benzofuran-2-yl(phenyl)methyl)-3-butyl-2-ethyl-1H-imidazol-3-ium iodide* on leukemia (HL-60), lung carcinoma (A549), colon carcinoma (SW480), breast carcinoma (MCF-7), and myeloid liver carcinoma (SMMC-7721) cancer lines have been detected over 40 *µ*M. Additionally, the IC_50_ activity of *3β-hydroxy-21-(1H-imidazol-1-yl)pregna-5,16-dien-20-one* on prostate cancer (PC-3), breast cancer (MCF7), and lung cancer (SK-LU- 1) were 20 *µ*M, 19 *µ*M, and 18 *µ*M, respectively [7]. These results are quantitatively better than 81 *µ*M and 57 *µ*M on Hep G2 and HeLa. However, this is compensated by the fact that the *L*_C_ and *L*_D_ have not any harmful effect on the healthy MEF cell lines.

## 4. Conclusions

Novel imidazole salts, or *N*-heterocyclic carbene ligands, namely, *1,3-bis(2-hydroxyethyl) imidazolidinium bromide L*_A_, *3-(2-ethoxy-2-oxoethly)-1-(3-aminopropyl)-1H-imidazol-3-ium bromide L*_B_, *1,3-bis(2-carboxyethyl)-4-methyl-1H-imidazol-3-ium bromide L*_C_, and *3-(2-carboxyethyl)-1-(3-aminopropyl)-1H-imidazol-3-ium bromide L*_D_ were synthesized, and they were preliminary confirmed by GC-MS and elemental analysis methods. Their molecular structures were theoretically determined, and they were confirmed by comparing calculated ^1^H, ^13^C NMR, and IR spectra with those of experimentally observed data. Also, the calculated structures were verified by the XRD results on a similar imidazole salt [22].

The antimicrobial and cytotoxic activities of the synthesized ligands on some specific bacteria and cancer cell lines were measured using spectrophotometric methods. It is seen that *L*_A_ showed better inhibition than the selected antibiotic on *Bacillus cereus* ATCC 11778 while it is effective on the selected bacteria and the yeast together with *L*_B_. On the cytotoxicity evaluation, *L*_C_ showed considerable inhibition effect on HeLa, as does *L*_D_ on Hep G2. Although their IC_50_ doses are quite high in comparison with the similar chemicals in the literature, the cytotoxicity of *L*_C_ and *L*_D_ is affirmed by not causing harmful effect on the healthy MEF cells as much as they do on the cancel cell lines.

## Figures and Tables

**Table 1:** Some selected geometrical parameters of the common properties of the investigated molecules.

	*L*_A_	*L*_B_	*L*_C_	*L*_D_	*L*
*Selected bond lengths* (Å)					
C_1_-H_1_	1.102	1.043	1.061	1.095	0.976
C_1_-N_1_	1.295	1.301	—	—	1.312 (7)
C_3_-N_1_	1.403	1.398	1.335	1.396	1.374 (8)
C_2_-C_3_	1.297	1.332	1.366	1.314	1.312 (10)
C_4_-N_2_	1.464	1.437	1.418	1.523	1.444 (8)
N_1_-C_5_	1.464	1.474	1.429	1.505	1.444 (8)
C_7B_-O_1B_	—	1.204	—	—	1.241 (8)
N_3B(D)_-H_3B(D)_	—	1.015	—	1.013	1.010

*Selected bond angles* (°)					
N_1_-C_1_-N_2_	108.7	112.6	111.7	108.9	109.2 (6)
C_1_-N_1_-C_3_	108.9	105.7	108.9	108.6	107.9 (5)
C_1_-N_1_-C_5_	125.1	127.6	126.0	126.0	126.2 (5)
C_4_-N_2_-C_1_	125.1	126.5	126.9	125.2	126.2 (5)
H_2_-C_2_-N_2_	122.6	121.8	121.8	122.3	123.4
C_3_-C_2_-H_2_	130.6	129.4	129.9	130.5	128.2

*Selected dihedral angles* (°)					
C_4_-N_2_-C_1_-N_1_	177.5	179.2	179.6	178.6	178.4 (5)
N_2_-C_2_-C_3_-N_1_	0.2	0.4	0.3	0.1	0.9 (8)
C_5_-N_1_-C_1_-N_2_	177.5	175.1	179.1	179.6	178.4 (5)
C_5_-N_1_-C_3_-C_2_	177.7	175.2	179.8	179.7	177.9 (6)

The data of the molecule showed by *L* in the last column reflect the X-ray diffraction measurements and calculated values on *1,3-bis(acetamide)imidazol-3-ium bromide* as given in [22]. The numbers in the paranthesis indicates the experimental error margins on the specific measurements. The labeled and numbered atoms in the first column are presented for each molecule in Scheme 1.

**Table 2:** Proton and carbon nuclear magnetic resonance spectral data of the molecules.

Assignment	Proton chemical shift (ppm)		Assignment	Carbon chemical shift (ppm)	
	Experimental	Theory		Experimental	Theory
*1,3-Bis(2-hydroxyethyl) imidazolidinium bromide* (*L*_A_)					
*s*, 1H, NCHN	9.06	9.06	NCHN	136.33	137.66
*d*, 2H, HC=CH	7.70	7.32	HC=CH	122.60	123.06
*t*, 4H, N-CH_2_	3.93	4.01	O-CH_2_	59.75	61.68
*t*, 4H, O-CH_2_	3.82	3.87	N-CH_2_	51.98	52.59
*s*. 2H, OH	—	1.73	—	—	—

*3-(2-Ethoxy-2-oxoethly)-1-(3-aminopropyl)-1H-imidazol-3-ium bromide* (*L*_B_)					
*s*, 1H, NCHN	7.79	7.73	CO	171.85	172.23
*d*, 1H, HC=CH	7.25	7.23	NCHN	136.78	137.10
*d*, 1H, HC=CH	7.02	7.00	HC=CH	125.13	124.74
*s*, 2H, N-CH_2_ CO	4.23	4.22 (2)	HC=CH	120.73	121.34
*q*, 2H, CH_2_-CH_3_	3.23	3.21 (3)	O-CH_2_-CH_3_	60.78	57.36
*t*, 2H, N-CH_2_-C	2.95	2.96 (3)	N-CH_2_	54.97	54.10
*t*, 2H, NH_2_-CH_2_	2.55	2.54 (2)	N-CH_2_-CH_2_-	44.97	38.64
*m*, 2H, C-CH_2_-C	2.20	2.18 (2)	NH_2_-CH_2_-	36.84	36.61
*t*, 3H, CH_3_	2.02	2.04 (3)	C-CH_2_-C	28.72	31.52
NH_2_	—	0.5 (3)	CH_3_	13.64	17.70

*1,3-Bis(2-carboxyethyl)-4-methyl-1H-imidazol-3-ium bromide* (*L*_C_)					
*s*, 1H, NCHN	8.84	8.83	COOH	173.03	170.3 (1)
*s*, 1H, HC=C-CH_3_	7.33	7.34	NCHN	133.30	132.96
*s*, OH	—	5.95 (2)	C-CH_3_	129.94	130.93
*t*, 4H, N-CH2	3.64	3.62 (5)	C=C-CH_3_	116.01	119.67
			N-CH_2_	37.53	39.7 (9)
*t*, 4H, CH_2_-COOH	2.91	3.10 (2)	CH_2_-COOH	26.79	33.8 (5)
*s*, 3H, -CH3	2.38	2.37	CH_3_	9.01	10.26

*3-(2-Carboxyethyl)-1-(3-aminopropyl)-1H-imidazol-3-ium bromide* (*L*_D_)					
*s*, 1H, NCHN	9.11	9.11	COOH	170.21	177.74
*d*, 1H, HC=CH	7.72	7.71	NCHN	137.16	137.47
*d*, 1H, HC=CH	7.65	7.63	HC=CH	123.94	124.97
*s*, OH	—	6.39	HC=CH	121.50	124.69
*t*, 4H, N-CH_2_	4.45	4.47 (1)	N-CH_2_	60.01	60.29
*t*, 2H, COOH-C	3.33	3.34	N-CH_2_	52.02	53.30
*t*, 2H, NH_2_-CH_2_	3.05	3.03	NH_2_-CH_2_	45.42	45.41
*m*, 2H, C-CH_2_-C	2.33	2.31	C-CH_2_-C	36.18	40.05
NH_2_	—	0.7 (3)	COOH-CH_2_	27.76	34.43

**Table 3:** The experimental and theoretical vibrational wave numbers for the infrared spectra of *1,3-bis(2-hydroxyethyl) imidazolidinium bromide* (*L*_A_) with its symbolled and numbered atoms in Figure 1.

Vibrational assignments/vibrating atoms	Theory	Experiment
*ν*_s_(OH)/(O_1A_-H_1A_, H_2A_-O_2A_)	3477	3358
*ν*_as_(OH)/(O_1A_-H_1A_, H_2A_-O_2A_)	3477	
*ν*_as_(CH_2_)/(C_4_-H_4_, H′_4_-C_4_), (C_5_-H_5_,H′_5_-C_5_)	3093	3114–2822
*ν*_as_(CH_2_)/(C_6A_-H_6A_, H′_6A_-C_6A_), (C_7A_-H_7A_, H′_7A_-C_7A_)	3011	
*ν*(NCHN)/(C_1_-H_1_)	2949	
*ν*_s_(CH)/(C_2_-H_2_, H_3_-C_3_)	2928	
*ν*_s_(CH_2_)/(C_4_-H_4_, H′_4_-C_4_), (C_5_-H_5_, H′_5_-C_5_)	2914	
*ν*_as_(CH)/(C_2_-H_2_, H_3_-C_3_)	2899	
*ν*_s_(CH_2_)/(C_6A_-H_6A_, H′_6A_-C_6A_), (C_7A_-H_7A_, H′_7A_-C_7A_)	2880	2808
*ν*_as_(CN)/(C_1_-N_1_,N_2_-C_1_)	1682	1630
*ν*_s_(CN)/(C_1_-N_1_,N_2_-C_1_)	1543	1592
*δ*_sc_(CH_2_)/(C_6A_-H_6A_, H′_6A_-C_6A_), (C_7A_-H_7A_, H′_7A_-C_7A_)	1452	1463
*δ*_sc_(CH_2_)/(C_4_-H_4_, H′_4_-C_4_), (C_5_-H_5_, H′_5_-C_5_)	1428	
*δ*(OH)/(O_1A_-H_1A_), (O_2A_-H_2A_)	1411	
*γ*_t_(CH_2_) + *δ*_r_(CH) + *δ*(NCHN)/(C_4_-H_4_, H′_4_-C_4_), (C_2_-H_2_, C_3_-H_3_), (C_1_-H_1_)	1375	1373
*γ*_w_(CH_2_) + *ν*_s_(CN)/(C_5_-H_5_, H′_5_-C_5_), (C_7A_-H_7A_, H′_7A_-C_7A_), (C_4_-N_2_, N_1_-C_5_)	1355	1330
*δ*_r_(CH)/(C_2_-H_2_, H_3_-C_3_)	1345	
*γ*_t_(CH_2_) + *δ*(NCHN) + *δ*_r_(CH)/(C_6A_-H_6A_,H′_6A_-C_6A_), (C_1_-H_1_), (C_2_-H_2_, H_3_-C_3_)	1323	1301
*γ*_t_(CH_2_) + *δ*(NCHN) + *δ*_r_(CH)/(C_4_-H_4_, H′_4_-C_4_), (C_1_-H_1_), (C_2_-H_2_, H_3_-C_3_)	1313	
*δ*(NCHN) + *γ*_w_(OH)/(C_1_-H_1_), (O_1A_-H_1A_, H_2A_-O_2A_)	1274	1265
*γ*_t_(CH_2_) + *δ*_r_(OH)/(C_4_-H_4_, H′_4_-C_4_), (C_5_-H_5_, H′_5_-C_5_), (O_1A_-H_1A_, O_2A_-H_2A_)	1212	
*γ*_t_(CH_2_) + *δ*_r_(OH)/(C_6A_-H_6A_, H′_6A_-C_6A_), (C_7A_-H_7A_, H′_7A_-C_7A_), (O_1A_-H_1A_, O_2A_-H_2A_)	1209	
*δ*_sc_(CH)/(C_2_-H_2_, H_3_-C_3_)	1149	1179
*ν*_as_(CN)/(C_3_-N_1_, N_2_-C_2_) + (C_4_-N_2_, N_1_-C_5_)	1143	
*ν*_as_(CO) + *ν*_as_(CC)/(C_6A_-O_2A_, O_1A_-C_7A_), (C_6A_-C_4_, C_5_-C_7A_)	1077	1080
*ν*_s_(CO) + *ν*_s_(CC)/(C_6A_-O_2A_, O_1A_-C_7A_), (C_6A_-C_4_, C_5_-C_7A_)	1076	
*δ*_r_(CH_2_) + *ν*_s_(CO)/(C_4_-H_4_, H′_4_-C_4_), (C_5_-H_5_, H′_5_-C_5_), (C_6A_-O_2A_, C_7A_-O_1A_)	1047	
*δ*_r_ (CH_2_) + *ν*_s_ (CN)/(C_6A_-H_6A_, H′_6A_-C_6A_), (C_7A_-H_7A_, H′_7A_-C_7A_), (C_3_-N_1_, N_2_-C_2_)	997	956
*γ*(NCHN)/(C_1_-H_1_)	946	
*ν*_s_(CC)/(C_6A_-C_4_, C_5_-C_7A_)	930	917
*δ*_r_(CH_2_) + *ν*_as_(CO)/(C_4_-H_4_, H′_4_-C_4_), (C_5_-H_5_, H′_5_-C_5_), (C_6A_-O_2A_, O_1A_-C_7A_)	867	875
*δ*_r_(CH_2_)/(C_4_-H_4_, H′_4_-C_4_), (C_6A_-H_6A_, H′_6A_-C_6A_)	861	
*γ*_w_(CH) + *γ*(NCHN)/(C_2_-H_2_, H_3_-C_3_), (C_1_-H_1_)	853	840
*ν*_as_(CN) + *δ*_r_(CH_2_)/(N_2_-C_4_, N_1_-C_5_), (C_6A_-H_6A_, H′_6A_-C_6A_), (C_7A_-H_7A_, H′_7A_-C_7A_)	677	673
*γ*(NCHN)/(C_1_-H_1_)	617	639

*ν*, stretching; *δ*, in-plane bending; *γ*, out-of-plane bending; s, symmetric; as, asymmetric; sc, scissoring; r, rocking; t, twisting; w, wagging.

**Table 4 tab4:** The experimental and theoretical vibrational wave numbers for the infrared spectra of *3-(2-ethoxy-2-oxoethly)-1-(3-aminopropyl)-1H-imidazol-3-ium bromide* (*L*_B_) with its symbolled and numbered atoms in Figure 1.

Vibrational assignments/vibrating atoms	Theory	Experiment
*ν*(NCHN)/(C_1_-H_1_)	3460	3400
*ν*_as_(NH_2_)/(N_3B_-H_3B_, H′_3B_-N_3B_)	3412	
*ν*_s_(NH_2_)/(N_3B_-H_3B_, H′_3B_-N_3B_)	3345	
*ν*_as_(CH_2_)/(C_9B_-H_9B_, H′_9B_-C_9B_)	3327	3192–2720
*ν*_s_(CH_2_) + *ν*_s_(CH_2_)/(C_1_-H_5_, H′_5_-C_1_), (C_9B_-H_9B_, H′_9B_-C_9B_)	3270	
*ν* (CH_2_)/(C_4_-H′_4_)	3263	
*ν* (CH_2_)/(C_8B_-H_8B_)	3226	
*ν*_as_(CH_2_)/(C_5_-H_5_, H′_5_-C_5_)	3221	
*ν*_s_(CH_2_)/(C_5_-H_5_, H′_5_-C_5_)	3157	
*ν*(CH)/(C_3_-H_3_)	3087	
*ν*(CH)/(C_2_-H_2_)	2946	
*ν*_as_(CH_2_)/(C_6B_-H_6B_, H′_6B_-C_6B_)	2843	
*ν*_s_(CH_2_)/(C_6B_-H_6B_, H′_6B_-C_6B_)	2795	
*ν*_as_(CH_3_)/(C_10B_-H_10B_, H′_10B_-C_10B_)	2784	
*ν*_as_(CH_3_)/(C_10B_-H_10B_, H″_10B_-C_10B_)	2783	
*ν*_s_(CH_3_)/(C_10B_-H_10B_, H′_10B_-C_10B_, H″_10B_-C_10B_)	2731	
*ν*(CH_2_)/(C_8B_-H′_8B_)	2606	2618
*ν*(CO)/(C_7B_-O_1B_)	1707	1753
*ν*(CN) + *δ*(CH) + *ν*(C=C)/(C_1_-N_2_), (C_2_-H_2_), (C_2_-C_3_)	1652	1645
*ν*(CN) + *ν*(C=C)/(C_1_-N_1_), (C_2_-C_3_)	1619	
*δ*_sc_(NH_2_)/(C_3B_-H_3B_, H′_3B_-C_3B_)	1606	
*ν*_s_(CN) + *ν*(NC)/(N_2_-C_1_,C_1_-N_1_), (N_2_-C_2_)	1494	1561
*δ*_sc_(CH_3_) + *δ*(CH_3_)/(C_10B_-H_10B_, C_10B_-H″_10B_), (C_10B_-H′_10B_)	1464	1528
*δ*_sc_(CH_2_)/(C_8B_-H_8B_, H′_8B_-C_8B_)	1462	
*δ*_sc_(CH_3_) + *δ*(CH_3_)/(C_10B_-H′_10B_, C_10B_-H″_10B_), (C_10B_-H_10B_)	1451	1473
*δ*_sc_(CH_2_)/(C_6B_-H_6B_, H′_6B_-C_6B_)	1441	
*δ*_sc_(CH_2_) + *γ*_w_(CH_3_)/(C_9B_-H_9B_, H′_9B_-C_9B_), (C_10B_-H_10B_, C_10B_-H′_10B_)	1415	1465
*δ*_sc_(CH_2_)/(C_5_-H_5_, H′_5_-C_5_)	1381	1458
*γ*_w_(CH_2_) + *δ*_sc_(CH_2_)/(C_8B_-H_8B_, H′_8B_-C_8B_), (C_4_-H_4_, H′_4_-C_4_)	1372	
*γ*_w_(CH_2_) + *δ*_sc_(CH_2_)/(C_6B_-H_6B_, H′_6B_-C_6B_), (C_4_-H_4_, H′_4_-C_4_)	1352	1394
*ν*_s_(CN) + *γ*_t_(CH_2_)/(C_4_-N_2_, N_1_-C_5_), (C_6B_-H_6B_, H′_6B_-C_6B_), (C_5_-H_5_, H′_5_-C_5_)	1319	1300
*γ*_w_(CH_2_)/(C_9B_-H_9B_, H′_9B_-C_9B_)	1309	
*γ*_t_(NH_2_) + *γ*_t_(CH_2_)/(N_3B_-H_3B_, H′_3B_-N_3B_), (C_6B_-H_6B_, H′_6B_-C_6B_)	1296	1242
*δ*(NCHN) + *δ*_r_(CH)/(C_1_-H_1_), (C_2_-H_2_, C_3_-H_3_)	1282	
*ν*_as_(CO) + *γ*_w_(CH_2_)/(C_7B_-O_2B_, O_2B_-C_9B_), (C_5_-H_5_, H′_5_-C_5_), (C_9B_-H_9B_, H′_9B_-C_9B_)	1248	1214
*ν*_as_(CO) + *γ*_w_(CH_3_)/(C_7B_-O_2B_, O_2B_-C_9B_), (C_10B_-H_10B_, C_10B_-H″_10B_)	1198	1183
*ν*_as_(CC) + *ν*(CO) + *ν*_s_(CN)/(C_8B_-C_6B_, C_6B_-C_4_), (C_3B_-O_2B_), (C_4_-N_2_, N_1_-C_5_)	1123	1118
*ν*_s_(CO) + *ν*(CC) + *δ*(NCHN)/(C_7B_-O_2B_, O_2B_-C_9B_), (C_9B_-C_10B_), (C_7B_-C_5_), (C_1_-H_1_)	1069	1097
*γ*_w_(NH_2_) + *δ*_r_(CH_2_)/(N_3B_-H_3B_, H′_3B_-N_3B_), (C_8B_-H_8B_, H′_8B_-C_8B_)	1011	1039
*γ*_w_(NH_2_) + *ν*(CC)/(N_3B_-H_3B_, H′_3B_-N_3B_) + (C_6B_-C_4_)	869	931
*ν*(CC) + *γ*_w_(CH_3_) + *ν*(CO)/(C_5_-C_7B_), (C_10B_-H_10B_, C_10B_-H′_10B_), (C_7B_-O_2B_)	860	852
*γ*_w_(NH_2_) + *ν*(CC)/(N_3B_-H_3B_, H′_3B_-N_3B_), (C_4_-C_6B_)	824	787
*γ*(NCHN) + *γ*_w_(CH)/(C_1_-H_1_), (C_2_-H_2_, C_3_-H_3_)	797	765
*γ*_w_(CH) + *γ*_t_(NH_2_) + *γ*_t_(CN)/(C_2_-H_2_, C_3_-H_3_), (N_3B_-H_3B_, H′_3B_-N_3B_), (N_1_-C_5_)	759	700
*γ*_t_(NH_2_) *γ*_t_(CN) + *γ*(NCHN)/(N_3B_-H_3B_, H′_3B_-N_3B_), (N_2_-C_4_), (C_1_-H_1_)	722	652
*γ*_t_(CH_2_) + *δ*(CC)/(C_8B_-H_8B_, H′_8B_-C_8B_), (C_6B_-H_6B_, H′_6B_-C_6B_), (C_5_-C_7B_)	665	636
*γ*_t_(CH_2_) + *δ*(CC)/(C_6B_-H_6B_, H′_6B_-C_6B_) + (C_6B_-C_4_) + (C_5_-C_7B_)	626	592
*γ*_t_(C=C)/(N_2_-C_2_, C_3_-N_1_)	603	
*Γ*(NCHN)/(C_1_-H_1_)	534	534

*ν*, stretching; *δ*, in-plane bending; *γ*, out-of-plane bending; s, symmetric; as, asymmetric; sc, scissoring; r, rocking; t, twisting; w, wagging.

**Table 5 tab5:** The experimental and theoretical vibrational wave numbers for the infrared spectra of *1,3-bis(2-carboxyethyl)-4-methyl-1H-imidazol-3-ium bromide* (*L*_C_) with its symbolled and numbered atoms in Figure 1.

Vibrational assignments/vibrating atoms	Theory	Experiment
*ν*(OH)/(O_3C_-H_3C_)	3685	3416
*ν*(OH)/(O_4C_-H_4C_)	3643	
*ν*(CH_2_)/(C_5_-H_5_)	3328	3268–2677
*ν*(CH_2_)/(C_4_-H_4_)	3311	
*ν*(NCHN)/(C_1_-H_1_)	3292	
*ν*(CH_3_)/(C_10_-H″_10C_)	3046	
*ν*(CH_2_)/(C_5_-H′_5_)	2983	
*ν*(CH_2_)/(C_4_-H′_4_)	2949	
*ν*_as_(CH_3_)/(C_10C_-H_10C_, H′_10C_-C_10C_)	2904	
*ν*_as_(CH_2_)/(C_7C_-H_7C_, H′_7C_-C_7C_)	2836	
*ν*_as_(CH_2_)/(C_6C_-H_6C_, H′_6C_-C_6C_)	2828	
*ν*_s_(CH_2_)/(C_6C_-H_6C_, H′_6C_-C_6C_)	2752	
*ν*_s_(CH_2_)/(C_7C_-H_7C_, H′_7C_-C_7C_)	2739	
*ν*(CH)/(C_2_-H_2_)	2706	2663
*ν*_s_(COOH) + *ν*_s_(OH)/(C_9C_-O_1C_, O_2C_-C_8C_), (O_3C_-H_3C_, H_4C_-O_4C_)	1785	1774
*ν*_as_(COOH) + *ν*_as_(OH)/(C_9C_-O_1C_, O_2C_-C_8C_), (O_3C_-H_3C_, H_4C_-O_4C_)	1783	
*ν*_as_(CN)/(C_1_-N_1_, N_2_-C_1_)	1756	
*ν*_as_(CN) + *ν*(CC) + *ν*(CN)/(C_1_-N_1_, N_2_-C_5_), (C_6C_-C_4_), (N_2_-C_4_)	1679	1662
*ν*_s_(CN) + *ν*(C=C) + *ν*(CC)/(C_1_-N_1_, N_2_-C_1_), (C_2_-C_3_), (C_3_-C_10C_), (C_4_-N_2_)	1642	
*γ*_t_(CH_2_) + *ν*(CN)/(C_5_-H_5_, H′_5_-C_5_), (N_1_-C_3_)	1510	1576
*γ*_t_(CH_2_) + *ν*(CN)/(C_4_-H_4_, H′_4_-C_4_), (N_2_-C_2_)	1476	1561
*δ*_sc_(CH_2_) + *δ*_sc_(CH_3_)/(C_4_-H_4_, H′_4_-C_4_), (C_6C_-H_6C_,H′_6C_-C_6C_), (C_10C_-H_10C_, C_10C_-H″_10C_)	1434	1468
*γ*(CH_3_)/(C_10C_-H′_10C_)	1398	1393
*δ*(CH) + *γ*_t_(CH_2_) + *δ*(NCHN)/(C_2_-H_2_) + (C_4_-H_4_, H′_4_-C_4_)+(C_5_-H_5_, H′_5_-C_5_) + (C_1_-H_1_)	1305	1317
*δ*(OH) + *ν*(CO)/(O_4C_-H_4C_), (C_8C_-O_4C_)	1259	1282
*δ*(CH) + *ν*(CC) + *ν*(CN)/(C_1_-H_1_), (C_2_-H_2_), (C_3_-C_10C_), (N_2_-C_4_)	1239	1235
*δ*(CH) + *ν*(CC) + *ν*_s_ (CN)/(C_1_-H_1_), (C_2_-H_2_), (C_3_-C_10C_), (N_2_-C_4_,C_5_-N_1_)	1221	
*γ*_t_(CH_2_)/(C_4_-H_4_, H′_4_-C_4_), (C_6C_-H_6C_, H′_6C_-C_6C_)	1191	1199
*γ*_t_(CH_2_)/(C_5_-H_5_, H′_5_-C_5_), (C_7C_-H_7C_, H′_7C_-C_7C_)	1181	
*γ*_w_(CH_2_) + *δ*(OH)/(C_6C_-H_6C_, H′_6C_-C_6C_), (O_4C_-H_4C_)	1149	1157
*γ*_w_(CH_2_) + *δ*(OH)/(C_7C_-H_7C_, H′_7C_-C_7C_), (O_3C_-H_3C_)	1139	
*ν*(CC)/(C_5_-C_7C_)	1086	1099
*ν*(CC)/(C_4_-C_6C_)	1074	
*δ*(CH_3_)/(C_10_-H_10C_, C_10_-H′_10C_, C_10_-H″_10C_)	1046	1031
*γ*(CH_3_) + *γ*(NCHN) + *γ*(CH)/(C_10_-H_10C_, C_10_-H'_10C_, C_10_-H″_10C_), (C_1_-H_1_), (C_2_-H_2_)	953	990
*γ*_w_(CH_2_) + *δ*_r_(CH_2_)/(C_6C_-H_6C_, H′_6C_-C_6C_), (C_4_-H_4_, H′_4_-C_4_)	940	931
*ν*(CC) + *δ*_r_(CH_2_)/(C_3_-C_10_), (C_7C_-C_9C_), (C_4_-H_4_, H′_4_-C_4_), (C_6C_-H_6C_, H′_6C_-C_6C_)	807	836
*γ*(NCHN)/(C_1_-H_1_)	786	
*γ*_t_(C=C) + *δ*(OH)/(N_2_-C_2_, C_3_-N_1_) + (O_4C_-H_4C_)	624	629
*δ*(OH)/(O_4C_-H_4C_)	470	462
*δ*(OH)/(O_3C_-H_3C_)	424	

*ν*, stretching; *δ*, in-plane bending; *γ*, out-of-plane bending; s, symmetric; as, asymmetric; sc, scissoring; r, rocking; t, twisting; w, wagging.

**Table 6:** The experimental and theoretical vibrational wave numbers for the infrared spectra of *3-(2-carboxyethyl)-1-(3-aminopropyl)-1H-imidazol-3-ium bromide* (*L*_D_) with its symbolled and numbered atoms in Figure 1.

Vibrational assignments/vibrating atoms	Theory	Experiment
*ν*(OH)/(O_2D_-H_2D_)	3590	3423
*ν*_as_(NH_2_)/(N_3D_-H_3D_, H′_3D_-N_3D_)	3446	
*ν*(CH_2_)/(C_4_-H_4_)	3381	3222–2666
*ν*_s_(NH_2_)/(N_3D_-H_3D_, H′_3D_-N_3D_)	3370	
*ν*(CH_2_)/(C_5_-H_5_)	3154	
*ν*(CH_2_)/(C_8D_-H_8D_)	3152	—
*ν*(NCHN)/(C_1_-H_1_)	2995	
*ν*(CH_2_)/(C_6D_-H_6D_)	2890	
*ν*_s_(CH)/(C_2_-H_2_, H_3_-C_3_)	2857	
*ν*_as_(CH_2_)/(C_7D_-H_7D_, H′_7D_-C_7D_)	2850	
*ν*_as_(CH) + *ν*_s_(CH_2_)/(C_2_-H_2_, H_3_-C_3_), (C_5_-H_5_, H′_5_-C_5_)	2828	
*ν*_s_(CH_2_)/(C_7D_-H_7D_, H′_7D_-C_7D_)	2789	
*ν*(CH_2_)/(C_4_-H′_4_)	2752	
*ν*(CH_2_)/(C_8D_-H′_8D_)	2709	
*ν*(COOH)/(C_9D_-O_1D_)	1728	1745
*ν*_s_(CN) + *ν*(C=C)/(C_1_-N_1_, N_2_-C_1_), (C_2_-C_3_)	1668	1642
*ν*_as_(CN) + *δ*_r_(CH)/(C_1_-N_1_, N_2_-C_1_), (C_2_-H_2_, H_3_-C_3_)	1601	1585
*δ*_sc_(NH_2_)/(N_3D_-H_3D_, H′_3D_-N_3D_)	1582	
*ν*_s_(CN)/(C_1_-N_1_, N_2_-C_1_)	1468	1470
*δ*_sc_(CH_2_)/(C_6D_-H_6D_, H′_6D_-C_6D_)	1454	
*δ*_sc_(CH_2_)/(C_8D_-H_8D_, H′_8D_-C_8D_)	1443	1419
*δ*_sc_(CH_2_) + *γ*_w_(CH_2_)/(C_5_-H_5_, H′_5_-C_5_), (C_7D_-H_7D_, H′_7D_-C_7D_)	1430	
*δ*_sc_(CH_2_)/(C_4_-H_4_, H′_4_-C_4_)	1423	
*γ*_w_(CH_2_) + *ν*(CC) + *ν*(CO)/(C_7D_-H_7D_, H′_7D_-C_7D_), (C_7D_-C_9D_) + (C_9D_-O_2D_)	1417	1354
*δ*_sc_(CH_2_)/(C_7D_-H_7D_, H′_7D_-C_7D_)	1404	
*δ*(NCHN) + *δ*_r_(CH)/(C_1_-H_1_), (C_2_-H_2_, H_3_-C_3_)	1372	
*γ*_w_(CH_2_) + *ν*(CC)/(C_4_-H_4_,H′_4_-C_4_), (C_6D_-H_6D_, H′_6D_-C_6D_), (C_6D_-C_8D_)	1369	
*γ*_w_(CH_2_) + *γ*_t_(NH_2_)/(C_4_-H_4_, H′_4_-C_4_), (C_6D_-H_6D_,H′_6D_-C_6D_), (N_3D_-H_3D_,H′_3D_-N_3D_)	1348	1318
*γ*_w_(CH_2_)/(C_5_-H_5_, H′_5_-C_5_), (C_7D_-H_7D_, H′_7D_-C_7D_)	1344	
*δ*_r_(CH) + *γ*_t_(CH_2_) + *γ*_w_(CH_2_)/(C_2_-H_2_, H_3_-C_3_), (C_5_-H_5_, H′_5_-C_5_), (C_8D_-H_8D_, H′_8D_-C_8D_)	1314	1297
*δ*(NCHN) + *δ*_r_(CH) + *δ*(OH)/(C_1_-H_1_), (C_2_-H_2_, H_3_-C_3_), (O_2D_-H_2D_)	1298	
*γ*_w_(CH_2_) + *δ*(OH)/(C_7D_-H_7D_, H′_7D_-C_7D_), (O_2D_-H_2D_)	1283	
*γ*_t_(NH_2_) + *γ*_t_(CH_2_)/(N_3D_-H_3D_, H′_3D_-N_3D_), (C_6D_-H_6D_, H′_6D_-C_6D_)	1266	1225
*γ*_t_(NH_2_)/(N_3D_-H_3D_, H′_3D_-N_3D_)	1209	
*δ*_sc_(CH) + *δ*(CH) + *γ*_t_(CH_2_)/(C_2_-H_2_, H_3_-C_3_), (C_1_-H_1_), (C_4_-H_4_, H′_4_-C_4_)	1178	1174
*δ*_sc_(CH)/(C_2_-H_2_, H_3_-C_3_)	1148	1119
*δ*(NCHN) + *γ*_w_(CH_2_) + *δ*(OH)/(C_1_-H_1_), (C_7D_-H_7D_,H′_7D_-C_7D_), (O_2D_-H_2D_)	1139	
*δ*(NCHN) + *γ*_t_(CH_2_) + *γ*_t_(NH_2_)/(C_1_-H_1_), (C_5_-H_5_, H′_5_-C_5_), (N_3D_-H_3D_, H′_3D_-N_3D_)	1111	1098
*γ*_t_(NH_2_) + *δ*_sc_(CH) + *ν*_as_(CN)/(N_3D_-H_3D_, H′_3D_-N_3D_), (C_2_-H_2_,H_3_-C_3_), (C_4_-N_2_, N_1_-C_5_)	1070	1069
*ν*(CC)/(C_5_-C_7D_)	1037	1033
*γ*_w_(NH_2_) + *ν*(CC) + *ν*(CN)/(N_3D_-H_3D_,H′_3D_-N_3D_), (C_6D_-C_4_), (C_4_-N_2_)	1034	
*γ*_t_(CH)/(C_2_-H_2_,H_3_-C_3_)	973	976
*γ*_w_(CH_2_) + *δ*_r_(CH_2_) + *δ*(OH)/(C_7D_-H_7D_,H′_7D_-C_7D_), (C_5_-H_5_, H′_5_-C_5_), (O_2D_-H_2D_)	960	
*γ*_t_(NH_2_) + *ν*(CC)/(N_3D_-H_3D_, H′_3D_-N_3D_), (C_6D_-C_8D_)	948	
*γ*(NCHN) + *γ*_w_(CH)/(C_1_-H_1_), (C_2_-H_2_,H_3_-C_3_)	918	954
*γ*_t_(NH_2_) + *ν*(CN) + *δ*_r_(CH_2_)/(N_3D_-H_3D_, H′_3D_-N_3D_), (N_3D_-C_8D_), (C_6D_-H_6D_, H′_6D_-C_6D_)	818	846
*ν*_s_(CC) + *ν*(CO) + *δ*(OH)/(C_5_-C_7D_,C_7D_-C_9D_), (C_9D_-O_2D_), (O_2D_-H_2D_)	801	767
*γ*_t_(C=C) + *δ*(OH)/(C_2_-C_3_), (O_2D_-H_2D_)	663	637
*δ*(OH) + *δ*_r_(CH_2_)/(O_2D_-H_2D_), (C_7D_-H_7D_, H′_7D_-C_7D_)	530	—

*ν*, stretching; *δ*, in-plane bending; *γ*, out-of-plane bending; s, symmetric; as, asymmetric; sc, scissoring; r, rocking; t, twisting; w, wagging.

**Table 7:** Half inhibition concentrations of the molecules on the selected bacteria and cancer cell lines.

	Antibacterial activity IC_50_ (*µ*M)			Cytotoxic activity IC_50_ (*µ*M)		
	*Escherichia coli* O157:H7	*Candida albicans*	*Bacillus cereus*	HeLa	Hep G2	MEF
*L*_A_	32	30	17	316	100	—
*L*_B_	39	156	29	141	182	—
*L*_C_	—	—	—	81	150	—
*L*_D_	—	—	—	167	57	—
Antibiotic	10	30	56	—	—	—

*L*_A_, 1,3-bis(2-hydroxyethyl) imidazolidinium bromide; *L*_B_, 3-(2-ethoxy-2-oxoethly)-1-(3-aminopropyl)-1H-imidazol-3-ium bromide; *L*_C_, 1,3-bis(2-carboxyethyl)-4-methyl-1H-imidazol-3-ium bromide; *L*_D_ 3-(2-carboxyethyl)-1-(3-aminopropyl)-1H-imidazol-3-ium bromide; IC_50_, half inhibitory concentrations; HeLa, human cervical cancer cell line; Hep G2, human liver cancer cell line; MEF, healthy mouse embryonic fibroblast cell line.

## Data Availability

The data used to support the findings of this study are available from the corresponding author upon request.
